# Emphysematous Cystitis in an Immunocompetent Patient on an SGLT2 Inhibitor

**DOI:** 10.7759/cureus.83680

**Published:** 2025-05-07

**Authors:** Kailey N Nguyen, Dipesh R Bista

**Affiliations:** 1 Graduate Medical Education, Baylor Scott & White All Saints, Fort Worth, USA; 2 Internal Medicine, Baylor Scott & White All Saints, Fort Worth, USA

**Keywords:** diabete mellitus, empagliflozin, emphysematous cystitis (ec), klebsiella pneumonea, non-immunocompromised host, sglt-2 inhibitors

## Abstract

Emphysematous cystitis is a rare, severe infection of the urinary bladder caused by gas-forming organisms like *Escherichia coli *and* Klebsiella pneumoniae *(*K. pneumoniae*). Common risk factors are female sex, chronic urinary infection, immunosuppression, diabetes mellitus, and neurogenic bladder. We report a case of *K. pneumoniae* emphysematous cystitis in a diabetic male patient being treated with empagliflozin. With this case report, we aim to highlight the potential role of an SGLT-2 inhibitor such as empagliflozin in developing severe urinary infections such as emphysematous cystitis.

## Introduction

Emphysematous cystitis (EC) is a rare infection of the urinary bladder associated with the presence of air within the bladder wall. The most common bacteria leading to emphysematous cystitis are *Escherichia coli* (*E. coli*) and* Klebsiella pneumoniae* (*K. pneumoniae*) [1]. Emphysematous cystitis is classically described in elderly females with a history of diabetes mellitus. Other risk factors have been described as chronic urinary tract infection, neurogenic bladder, long-term indwelling urinary catheter, and immunosuppression [2]. Sodium-glucose cotransporter 2 (SGLT2) inhibitor, such as empagliflozin, is a class of anti-diabetic medication that inhibits the sodium-glucose transporter from reabsorbing glucose from the lumen of the proximal convoluted tubules, leading to increased glucose excretion in the urine and lowered serum glucose. Here we present a case of EC in a diabetic 76-year-old male without prior history of urinary tract infection who had been treated with empagliflozin for diabetes mellitus. Our literature review showed that this is only the second case report suggesting a link between EC and an SGLT2 inhibitor such as empagliflozin.

## Case presentation

The patient is a 76-year-old male with past medical history significant for type 2 diabetes mellitus, hypertension, coronary artery disease, peripheral artery disease, bilateral below-knee amputation, and chronic obstructive pulmonary disease not currently on steroids who presented for evaluation of two weeks of dysuria and three days of hematuria described as “blood spotting in the urine.” The patient also endorsed lower back pain and suprapubic abdominal pain that started nine days prior and had since dissipated. He also reported gas coming out of his urethra after urination.

Home medications included empagliflozin, insulin detemir, insulin lispro, atorvastatin, ferrous sulfate, losartan-hydrochlorothiazide, vitamin B12, and vitamin D3.

Vital signs were all within normal limits except for elevated blood pressure (Table 1). Physical exam was only notable for suprapubic tenderness and absent costovertebral angle tenderness.

**Table 1:** Vital signs of the patient Patient's vital signs were within normal range except for high blood pressure.

Vitals	Patient	Normal Range
Blood pressure	176/76	<120/80 mmHg
Pulse rate	74	60-100 beats per minute
Respiratory rate	16	12-20 breaths per minute
Arterial oxygen saturation	97% on room air	95-100%

Urine analysis was significant for elevated glucose levels and findings consistent with urinary tract infection (Table 2).

**Table 2:** Urine analysis results The urinalysis result shows elevated urinary glucose levels and findings consistent with urinary tract infection. S1: slight cloudiness.

Markers	Patient	Normal Range
Turbidity	S1 Cloudy	Clear
pH	6.0	5.0-8.0
Protein	2+	Negative
Glucose	3+	Negative
Blood	3+	Negative
Leukocyte esterase	2+	Negative
Nitrites	Negative	Negative
Bilirubin	Negative	Negative
Urobilinogen	Negative	Negative
Ketones	Trace	Negative
Urine microscopy		
White blood cells	100+	0-5 per high power field
Red blood cells	100+	0-3 per high power field
Bacteria	Light	Absent
Epithelial cells	Rare	Few per high power field

Blood tests were also performed. A comprehensive metabolic panel was significant for elevated glucose levels; serum creatinine and blood urea nitrogen levels were also elevated, but they appeared to be the patient's baseline. Comprehensive blood count with differential was within normal limits (Table 3). Hemoglobin A1c level was found to be 9.7. 

**Table 3:** Blood test results Comprehensive metabolic panel was significant for elevated blood glucose level; serum creatinine and blood urea nitrogen were elevated but appeared to be the patient's baseline. Complete blood count results were within normal range.

Blood Test Results	Patient	Normal Range
Comprehensive metabolic panel		
Glucose	208	70–99 mg/dL
Blood urea nitrogen	30	7–18 mg/dL
Creatinine	1.79	0.70–1.30 mg/dL
Sodium	138	136–145 meq/L
Potassium	4.0	3.6–5.0 meq/L
Chloride	106	98–107 meq/L
Carbon dioxide	27	21–32 meq/L
Calcium	8.9	8.5–10.1 mg/dL
Total bilirubin	0.3	0.0–11.1 mg/dL
Alkaline phosphatase	120	40–129 U/L
Aspartate aminotransferase	24	0–40 U/L
Alanine aminotransferase	30	0–41 U/L
Total protein	6.3	6.1–7.9 g/dL
Albumin	3.0	3.9–5.2 g/dL
Complete blood count		
White blood cell	10.1	3.7–10.3 k/µL
Red blood cell	4.66	4.50–6.00 M/µL
Hemoglobin	13.6	13.5–18.0 g/dL
Hematocrit	41.0	40.0–52.0%
Mean corpuscular volume	88.0	80.0–99.0 fL
Mean corpuscular hemoglobin concentration	33.2	27.0–33.0 pg
Platelet	198	140–440 k/µL
Mean platelet volume	10.3	8.5–12.0 fL

Computed tomography (CT) scan of the abdomen and pelvis without contrast showed severe emphysematous cystitis, with bladder gas dissecting into peri-cystic fat, and an under-distended bladder. There was no hydronephrosis, and the prostate was noted to be mildly enlarged.

**Figure 1:**
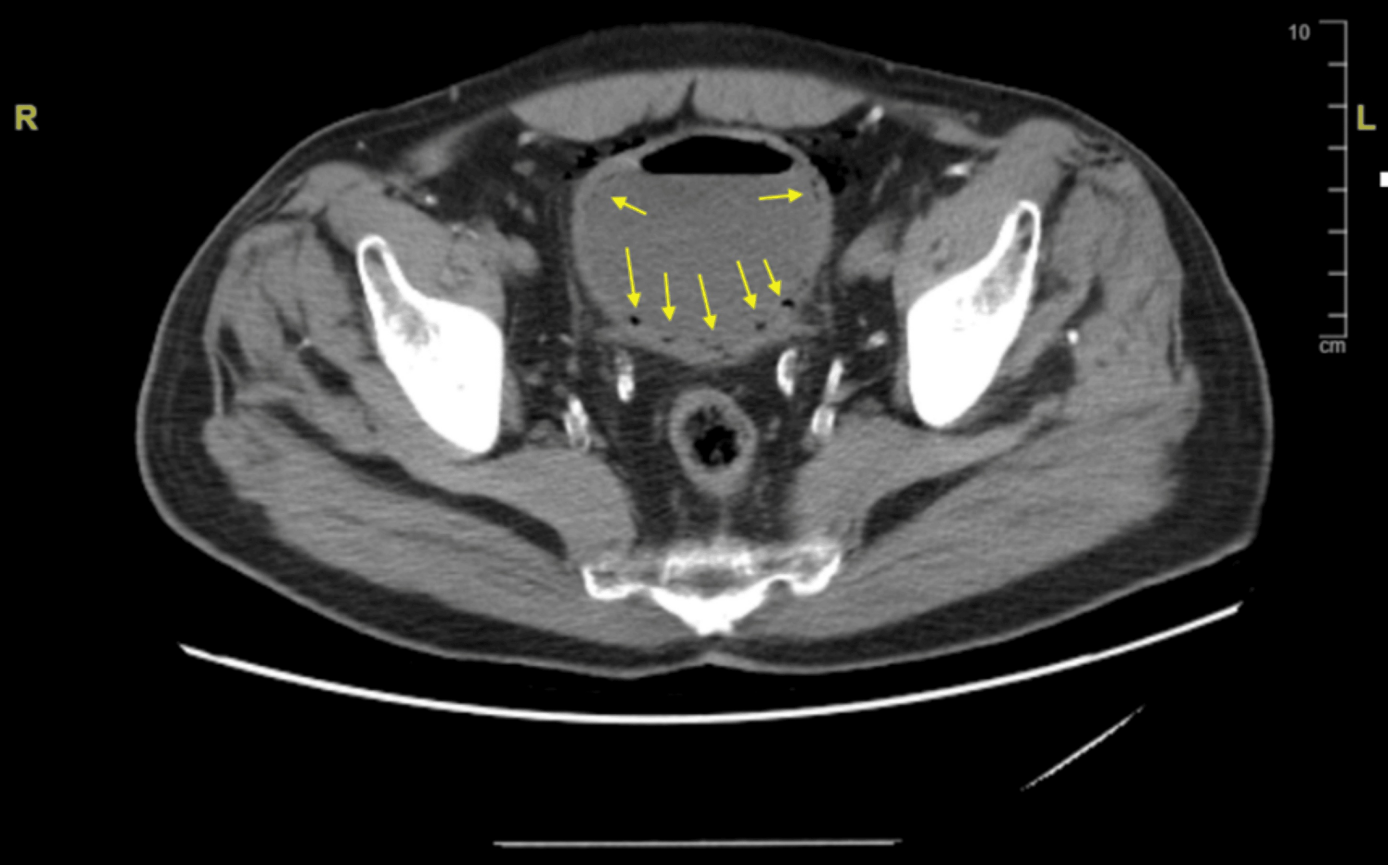
Computed tomography of the abdomen and pelvis The presence of air (yellow arrows) within the urinary bladder wall demonstrates findings consistent with emphysematous cystitis.

The patient was diagnosed with emphysematous cystitis and treated empirically with piperacillin-tazobactam, then transitioned to ceftriaxone given the lack of overt multidrug resistance risk factors. Urine culture eventually grew *Klebsiella pneumoniae*. On discharge, the patient was switched to oral ciprofloxacin according to sensitivity results to complete a three-week course of antibiotics. Empagliflozin was held on admission and later discontinued upon discharge due to concerns that it was exacerbating the infection via glycosuria.

## Discussion

Emphysematous cystitis is a rare complication of urinary tract infection. It is caused by organisms such as *Enterobacter aerogenes, Proteus mirabilis, Staphylococcus aureus, Streptococci, Clostridium perfringens, Candida albicans*, but most commonly *E. coli* and *K. pneumoniae* [1]. It is suggested that glucose fermentation by these bacteria forms gas within the bladder, leading to the characteristic finding of gas penetration into the bladder wall. Risk factors include old age and female sex, diabetes mellitus, immunosuppressant, neurogenic bladder, recurrent urinary tract infections [1,3]. Interestingly, classic urinary tract infection (UTI) symptoms, such as dysuria, increased frequency, and urgency, are not always present. A case series consisting of 53 cases reported that only about 53.3% of cases had classical UTI symptoms. More common presenting symptoms were abdominal pain, noted in 65.6% of cases, and hematuria, which was noted in 82.3% of cases [4]. Emphysematous cystitis presents on a wide spectrum ranging from asymptomatic to severe life-threatening illness, with the most severe cases associated with emphysematous pyelonephritis and urosepsis [5]. Mortality associated with EC has been reported to be between 1.78% to 7%, but increased to 14% when associated with another emphysematous infection of the urinary tract, such as emphysematous pyelonephritis [5,6]. The diagnostic method of choice is a CT scan, checking for the presence of gas within the bladder wall. Treatment ranges from antibiotics, which could be oral for mild, uncomplicated cases, to intravenous antibiotics, which are necessary in most cases. Up to 10% of cases have been reported to need surgical intervention like partial cystectomy, total cystectomy, or surgical debridement [5].

Sodium-glucose cotransporter 2 (SGLT2) inhibitors such as empagliflozin are being increasingly used in patients with type 2 diabetes mellitus due to their cardiovascular and reno-protective effects [7]. They inhibit sodium-glucose co-transporter-2, which under normal conditions reabsorbs filtered glucose in the proximal convoluted tubules. Thus, SGLT2 inhibitors allow more glucose to be excreted in the urine, leading to higher-than-normal levels of glucose in the urine. Huang et al. proposed that, along with the presence of gas-forming bacteria and impaired tissue perfusion, glycosuria is one of the three conditions thought to be critical in developing EC [3].

In their review analysis of thirteen trials of different SGLT2 inhibitors, Kittipubul et al. concluded that although SGLT2 inhibitors such as empagliflozin cause glycosuria, they do not increase the risk of urinary tract infections [8]. However, it is worth noting that in 2015, the Food and Drug Administration (FDA) issued a warning about the increased risk of severe UTI associated with SGLT2 inhibitors due to nineteen cases of life-threatening urosepsis and pyelonephritis reported within eighteen months since the approval of canagliflozin, the first SGLT2 inhibitor [9]. In 2018, the FDA issued another warning about the increased risk of necrotizing perineal infection requiring surgical debridement in patients taking SGLT2 inhibitors. This risk is estimated to be 200% higher with SGLT2 inhibitors than with other anti-diabetic drug classes [10]. Thus, we reckon the plausibility of increased risk of severe infection like EC in patients taking SGLT 2 inhibitors. Additionally, although the consensus is that SGLT2 inhibitors should be discontinued immediately upon the occurrence of severe infection [11,12], there is no established guidance on when and if SGLT2 inhibitors should be restarted in these patients after the infection has been treated. In our literature review, we also found one other case report of EC potentially being linked to SGLT2 inhibitor use [13].

## Conclusions

Emphysematous cystitis is a rare and potentially fatal infection of the urinary bladder, classically presenting in elderly diabetic females. We presented a case of emphysematous cystitis in a male patient with diabetes mellitus taking an SGLT2 inhibitor. Our patient only presented with mild symptoms such as dysuria and hematuria. He did not experience more severe complications, such as bladder perforation, and was managed non-surgically with intravenous antibiotics followed by a prolonged course of oral antibiotics for three weeks. In our literature review, a meta-analysis suggested that being on an SGLT2 inhibitor does not increase the risk of UTI, but on the other hand, there have been FDA warnings on increased risk of severe urinary tract infection as well as necrotizing perineal infection. Therefore, it is plausible to assume that taking an SGLT2 inhibitor increased the risk of more severe infection, like emphysematous cystitis, in this patient with uncontrolled diabetes mellitus. Given expanding indications for SGLT2 inhibitor use in patients with and without diabetes mellitus, we reckon more research is needed to investigate the role of SGLT2 inhibitors in the development of severe urinary tract infections to help further guide our management.
